# Research Ethics and Reporting Standards at *PLoS Neglected Tropical Diseases*

**DOI:** 10.1371/journal.pntd.0000069

**Published:** 2007-10-31

**Authors:** Gavin Yamey

*PLoS Neglected Tropical Diseases* has been open to submissions since February 2007, and we have been impressed by the number, quality, and range of the research articles we have received to date—from laboratory studies and assessments of new diagnostic tests to observational epidemiology, clinical trials, and qualitative research. But we have also noted that some authors have omitted crucial information about the ethical conduct of their study, or have been unaware of international guidance on how to report specific types of research. We would therefore like to remind authors of our reporting requirements (more detailed guidance is available at http://www.plosntds.org/guidelines.php).

## Ethical Approval and Informed Consent

Patients with neglected tropical diseases are among the most economically and socially disadvantaged people on the planet. So when it comes to conducting clinical research among this vulnerable population, investigators must take particular precautions to protect the welfare of research participants.

One crucial precaution that all researchers must take is to seek prior approval for their study from a relevant institutional review board (IRB), which must be named in the paper. We reserve the right to ask authors to send a copy of the board's formal approval. During the study, if the authors deviated from their protocol—and particularly if the deviation exposed participants to any additional risks—we expect the researchers to have immediately informed the board about the deviation and we require documentation of the board's response.

Some authors have argued that their study was exempt from IRB approval since the data were collected as part of the routine diagnosis or treatment of patients. But because of the potential risks involved in recording data about patients for research purposes, such as breaches of patient confidentiality, an IRB should be asked to assess these risks ahead of the study. The IRB may indeed decide that no specific approval is required, in which case we ask authors to send a copy of the IRB exemption letter.

We are aware that in some research settings there may not be a local IRB. Under these circumstances, authors should have made every attempt to find an ethics committee outside the immediate region to approve the study. In the very rare event that it was impossible to find such a committee, we expect authors to have conducted their study according to the principles expressed in the Declaration of Helsinki (http://www.wma.net/e/policy/b3.htm) and to have familiarized themselves with accepted international norms and standards, such as those set out by the International Committee of Medical Journal Editors (ICMJE; http://www.icmje.org/) and the Committee on Publication Ethics (http://www.publicationethics.org.uk/).

A second crucial precaution is that investigators must obtain fully informed, ideally written, consent from all participants in their study. We reserve the right to ask authors to send a copy of the consent form that the participants signed. If details about a specific patient are included in a paper (e.g., a case description or a clinical photograph), authors must send a copy of the patient's written informed consent to have these clinical details published in *PLoS Neglected Tropical Diseases* (using the consent form at http://www.plosntds.org/guidelines.php#supporting, available in English, French, Spanish, and Portuguese).

Oral consent is acceptable provided three specific conditions are met: (1) authors must explain in their paper why written consent was impossible (e.g., their study was conducted among an illiterate population); (2) authors must have documentary proof that every individual gave consent (e.g., a literate witness, who ideally was not part of the research team, signed on behalf of the participant, and/or there is an audio or video recording of the oral consent process); and (3) the IRB specifically approved the oral informed consent process [1]. A forthcoming policy article in *PLoS Neglected Tropical Diseases* explores in more detail the issue of obtaining meaningful and valid consent from vulnerable populations [2].

For animal studies, we ask authors to name the committee that approved the study and oversaw animal welfare.

## Reporting Guidelines

One of the most important advances in medical research reporting has been the development of international standards for reporting specific study types. PLoS has adopted these standards for all of its journals. The best known is the CONSORT (Consolidated Standards of Reporting Trials) statement. Authors should supply a completed CONSORT checklist (available from the CONSORT Web site at http://www.consort-statement.org/) and ensure that they include a flow diagram to illustrate participant flow through the trial. In addition, we strongly encourage authors to write their paper using a template that adopts the CONSORT section headings, and we have prepared such a template for you to use (available at http://www.plosone.org/static/tpl_plos_clinicaltrials.doc).

Authors of *all* trials (i.e., any study that prospectively assigns participants to one or more interventions) should send the original trial protocol as a supporting information file to allow editors and reviewers to assess the study fully. Any deviation from the trial protocol must be explained. We fully support the position of the ICMJE on trial registration [3]. All trials initiated after July 1, 2005 must be registered prospectively in a publicly accessible registry (before patient recruitment has begun), or they will not be considered for publication. For trials initiated before July 1, 2005, all trials must be registered before submission to our journals. The trial's registration number must be provided at the time of submission. Acceptable registries are listed on the World Health Organization's trials registration Web site (http://www.who.int/ictrp/network/list_registers/en/index.html). Further details on trial registration are available on the ICMJE's Web site (http://www.icmje.org/).

There are four other reporting standards that we ask authors to adopt. Systematic reviews should follow the QUOROM (Quality of Reports of Meta-Analyses) statement [4], and authors should include the QUOROM flow chart and checklist. Any study that reports on a diagnostic test should conform to the STARD requirements (http://www.consort-statement.org/index.aspx?o). Studies of observational epidemiology (cross-sectional, cohort, or case-control) should follow the STROBE (Strengthening the Reporting of Observational Studies in Epidemiology) statement (http://www.strobe-statement.org/). Finally, reports of microarray experiments should conform to the MIAME (Minimum Information About a Microarray Experiment) guidelines (http://www.mged.org/Workgroups/MIAME/miame.html) and the data from the experiments must be deposited in a publicly accessible database such as GEO (http://www.ncbi.nlm.nih.gov/geo/) or Array Express (http://www.ebi.ac.uk/arrayexpress/). Indeed, for all studies submitted to *PLoS Neglected Tropical Diseases*, all appropriate datasets, images, and information should be deposited in a relevant public database and the accession numbers should be given in the paper.

*PLoS Neglected Tropical Diseases* is committed to publishing research that not only advances scientific knowledge on these diseases of poverty, but that is ethically sound and reported to the very highest standards. Please help us in our mission by following our guidance on ethics and reporting standards.
